# *SULF1*作为特发性肺纤维化与肺腺癌共同基因的鉴定及其生物学功能分析

**DOI:** 10.3779/j.issn.1009-3419.2023.101.25

**Published:** 2023-09-20

**Authors:** Junyi WANG, Lu LU, Xiang HE, Lijuan MA, Tao CHEN, Guoping LI, Haijie YU

**Affiliations:** ^1^999078 澳门，澳门科技大学中药质量研究国家重点实验室 埃尔文内尔博士生物物理与创新药物实验室; ^1^Dr. Neher's Biophysics Laboratory for Innovative Drug Discovery, State Key Laboratory of Quality Research in Chinese Medicine, Macau University of Science and Technology, Macau 999078, China; ^2^610031 成都，成都市第三人民医院（重庆医科大学附属成都第二临床学院）呼吸与危重症医学科; ^2^Department of Pulmonary and Critical Care Medicine, Chengdu Institute of Respiratory Health, The Third People’s Hospital of Chengdu (The Second Clinical Medical College of Chongqing Medical University in Chengdu), Chengdu 610031, China; ^3^510000 广州，广州医科大学第一附属医院，呼吸疾病国家重点实验室; ^3^State Key Laboratory of Respiratory Disease, National Center of Respiratory Medicine, The First Affiliated Hospital of Guangzhou Medical University, Guangzhou 510000, China; ^4^529500 阳江，阳江市人民医院呼吸内科; ^4^Department of Respiratory Medicine, People’s Hospital of Yangjiang, Yangjiang 529500, China

**Keywords:** 肺肿瘤, 特发性肺纤维化, 加权基因共表达网络分析, SULF1, 单细胞转录组学分析, Lung neoplasms, Idiopathic pulmonary fibrosis, Weighted gene co-expression network analysis, SULF1, Single-cell RNA sequencing

## Abstract

**背景和目的** 特发性肺纤维化（idiopathic pulmonary fibrosis, IPF）是一种原因不明的慢性、进行性、间质性肺疾病，确诊后中位生存期为3-5年。IPF与肺癌风险增加有关。因此，探索IPF和肺腺癌的关键共同致病基因和分子通路，对开发IPF合并肺腺癌的新治疗手段和个性化精准治疗策略的制定具有重要意义。**方法** 利用基因表达综合（Gene Expression Omnibus, GEO）数据库中公开的IPF和肺腺癌基因表达数据集进行生物信息学分析。使用加权基因共表达网络分析识别涉及两种疾病进程的共同基因，进而功能富集分析。随后，联合额外数据集鉴定两种疾病的核心共同基因。并通过癌症基因组图谱计划（The Cancer Genome Atlas, TCGA）数据库和单细胞RNA测序数据集，分析核心共同基因与患者预后的关系，并评估其在肺腺癌中的表达模式、临床相关性、遗传特征和免疫相关功能。最后通过药物数据库筛选出相关的潜在治疗药物。**结果** 两者之间有529个共同致病基因。其中，SULF1作为核心共同致病基因与患者预后不良相关，其在肺腺癌组织中的表达水平显著升高，同时与高突变频率、显著基因组异质性以及抑制性免疫微环境相关。随后的单细胞分析发现SULF1高表达主要源于肿瘤相关成纤维细胞。SULF1表达与肿瘤药物敏感性变化相关，并筛选出与靶向SULF1高表达成纤维细胞相关的潜在小分子药物。**结论** 本研究鉴别出IPF和肺腺癌之间的共同分子途径和核心基因，其中SULF1可能作为两种疾病的潜在生物标志物和治疗靶点。

## Abstract (en)

**Background and objective** Idiopathic pulmonary fibrosis (IPF) is an idiopathic chronic, progressive interstitial lung disease with a diagnosed median survival of 3-5 years. IPF is associated with an increased risk of lung cancer. Therefore, exploring the shared pathogenic genes and molecular pathways between IPF and lung adenocarcinoma (LUAD) holds significant importance for the development of novel therapeutic approaches and personalized precision treatment strategies for IPF combined with lung cancer. **Methods ** Bioinformatics analysis was conducted using publicly available gene expression datasets of IPF and LUAD from the Gene Expression Omnibus (GEO) database. Weighted gene co-expression network analysis was employed to identify common genes involved in the progression of both diseases, followed by functional enrichment analysis. Subsequently, additional datasets were used to pinpoint the core shared genes between the two diseases. The relationship between core shared genes and prognosis, as well as their expression patterns, clinical relevance, genetic characteristics, and immune-related functions in LUAD, were analyzed using The Cancer Genome Atlas (TCGA) database and single-cell RNA sequencing datasets. Finally, potential therapeutic drugs related to the identified genes were screened through drug databases. **Results** A total of 529 shared genes between IPF and LUAD were identified. Among them, SULF1 emerged as a core shared gene associated with poor prognosis. It exhibited significantly elevated expression levels in LUAD tissues, concomitant with high mutation rates, genomic heterogeneity, and an immunosuppressive microenvironment. Subsequent single-cell RNA-seq analysis revealed that the high expression of SULF1 primarily originated from tumor-associated fibroblasts. This study further demonstrated an association between SULF1 expression and tumor drug sensitivity, and it identified potential small-molecule drugs targeting SULF1 highly expressed fibroblasts. **Conclusions ** This study identified a set of shared molecular pathways and core genes between IPF and LUAD. Notably, SULF1 may serve as a potential immune-related biomarker and therapeutic target for both diseases.

特发性肺纤维化（idiopathic pulmonary fibrosis, IPF）是一种原因不明的慢性、进行性间质性肺疾病。在特发性间质性肺炎中，IPF发病率最高，且仍在攀升^[1]^。IPF患者的生活质量显著下降，预后差，中位生存时间为3-5年^[2,3]^。IPF的典型临床病理特征为肺成纤维细胞活化，细胞外基质（extracellular matrix, ECM）过度积累，进而导致肺结构的不可逆损伤，最终因气体交换障碍而引起呼吸衰竭。

更令人担忧的是，IPF与患肺癌的风险增加相关。IPF患者肺癌风险相较正常人群增高5倍^[4]^。流行病学数据^[5]^显示IPF患者肺癌的发病率介于3%-22%，极端情况下甚至超过50%。值得注意的是，IPF患者的肺肿瘤往往首先出现在纤维化区域，推测这两种疾病之间可能存在共同的致病机制和分子通路。同时，与非IPF相关的肺癌相比，IPF相关肺癌在组织学分布和免疫组化特征上均表现出明显不同^[4]^，引发了关于如何更有效地治疗和管理IPF合并肺癌患者的思考。因此，揭示IPF和肺肿瘤之间的共同致病机制，并由此探索潜在靶向分子，可使我们更好地理解IPF增加肺癌风险的分子机制，掌握IPF的演进和转归，对IPF基础上肺肿瘤新治疗手段的开发和个性化精准治疗策略的制定具有重要意义。

## 1 资料与方法

### 1.1 数据收集和处理

IPF（GSE150910、GSE53845和GSE32537）和肺腺癌（lung adenocarcinoma, LUAD）（GSE32863和GSE68465）数据集从基因表达综合（Gene Expression Omnibus, GEO）数据库（http://www.ncbi.nlm.nih.gov/geo/）获取。其中，GSE150910数据集包括103例IPF和103例对照肺组织的RNA测序数据。GSE53845数据集包含40例IPF患者和8例对照组织的基因表达芯片数据。GSE32537包括119例IPF和50例对照肺组织的基因表达芯片数据。GSE32863数据集包括58例LUAD样本和58例正常邻近组织样本的RNA测序数据。GSE68465数据集包含433例LUAD样本和19例对照组织的基因表达芯片数据。此外，我们从Code Ocean平台（https://codeocean.com/capsule/8321305/tree）下载了6例LAUD初治手术患者肿瘤组织及其配对正常肺实质组织共12个样本的单细胞RNA测序数据^[6]^。

### 1.2 加权基因共表达网络分析（weighted gene co-expression network analysis, WGCNA）

WGCNA是一种广泛应用于分析大规模数据集的方法，可以识别与疾病进展密切相关的一组基因。本研究使用R包“WGCNA”（1.72-1）通过hclust()聚类，转换邻接矩阵（softPower=4），TOM矩阵TOMsimilarity()、cutreeDynamic()识别动态剪切模块、mergeCloseModules()合并相似模块等构建基因共表达网络^[7]^。

### 1.3 鉴定IPF和LUAD之间共享的基因

本研究使用Venny 2.1.0获得模组间的交集基因。随后使用STRING数据库（https://string-db.org/）构建了蛋白质相互作用（protein-protein interaction, PPI）网络。

### 1.4 功能富集分析

PPI网络的功能富集使用Cytoscape软件ClueGO插件进行，余下差异基因使用R包“clusterProfiler（4.8.3）” ^[8]^进行富集分析。

### 1.5 差异表达基因分析

主成分分析（principal component analysis, PCA）质控样本后，使用R中的“limma（3.56.2）”包^[9]^进行标准化校正和差异表达基因分析。GSE68465中，阈值为|log2 fold change|≥2和P<0.05。而在GSE53845中，为|log2 fold change|≥1和P<0.05。最后利用Venny 2.1.0进行交集分析。

### 1.6 基于基因表达水平值的交互式分析（gene expression profiling interactive Analysis, GEPIA）数据库

GEPIA数据库收集了来自癌症基因组图谱计划（The Cancer Genome Atlas, TCGA）和基因型-组织表达（Genotype-Tissue Expression, GTEx）数据集的9736个肿瘤组织和8587个正常组织的RNA测序数据，本研究使用GEPIA数据库1.0版本（http://gepia.cancer-pku.cn/index.html）^[10]^中Expression DIY功能模块评估TCGA-LUAD数据集SULF1的表达。

### 1.7 阿拉巴马大学癌症数据分析（The University of ALabama at Birmingham CANcer data analysis Portal, UALCAN）数据库

本研究使用UALCAN数据库（http://ualcan.path.uab.edu/）^[11]^（更新于2022-08-16），使用TCGA功能模块分析TCGA转录组学数据集中LUAD的SULF1表达及其与组织类型、性别、年龄、肿瘤分期、淋巴结转移状态、TP53突变状态和启动子甲基化水平等多种临床病理参数的关联。使用Proteomics功能模块分析临床蛋白质组肿瘤分析协作组（Clinical Proteomic Tumor Analysis Consortium, CPTAC）数据集中LUAD的SULF1蛋白表达。

### 1.8 Kaplan-Meier生存分析

使用KM Plotter（http://kmplot.com）^[12]^（更新于2023-04-18）的Start KM Plotter for lung cancer功能模块通过患者SULF1表达中位数分层评估在LUAD中的预后价值。

### 1.9 受试者工作特征曲线分析

受试者工作特征（receiver operator characteristic, ROC）曲线分析使用R包“pROC（1.18.4）”完成。

### 1.10 肿瘤免疫治疗多组学综合分析（Comprehensive Analysis on Multi-Omics of Immunotherapy in Pan-cancer, CAMOIP）数据库

使用CAMOIP数据库（http://camoip.net/）^[13]^（更新于2022-04-11）中的TCGA-LUAD数据，以患者SULF1表达高低分组，通过Mutational Landscape功能模块进行基因突变谱分析，Immune Infiltration功能模块进行免疫浸润分析，Immunogenicity功能模块进行免疫原性分析，Pathway Enrichment功能模块进行基因集富集分析。

### 1.11 单细胞RNA测序数据分析

使用R包“Seurat（4.0）”筛选高质量细胞（500 to 7500 genes, 1000 to 50,000 UMIs, mitochondria content less than 30%, and HB less than 5%）^[14]^。“NormalizeData”函数进行数据归一化，并通过“FindVariableFeatures”函数识别高变基因。“FindIntegrationAnchors”和“IntegrateData”函数整合数据，“FindClusters”进行主成分分析和聚类（30 PCs/resolution: 0.8）。使用“RunTSNE”或“RunUMAP”函数降维。使用“FindMarkers”和“FindAllMarkers”函数分析细胞亚群之间的差异表达基因（min.pct=0.3, logfc.threshold=0.25）^[15]^。

### 1.12 药物敏感性分析

从CellMiner（https://discover.nci.nih.gov/cellminer/home.do）数据库中获取基因表达谱数据和美国国家癌症研究所60个人类肿瘤细胞系抗癌药物筛选化合物数据。本研究纳入了CellMiner数据库中75种经过临床试验和188种经过美国食品药品监督管理局（Food and Drug Administration, FDA）批准的共263个药物，使用“impute（1.74.1）”“limma”“ggplot2（3.4.3）”和“ggpubr（0.6.0）”等R包，通过Pearson算法，以P<0.05和|cor|>0.3为阈值，分析SULF1基因对药物敏感性的影响^[16]^。

### 1.13 免疫治疗反应的预测

使用肿瘤免疫功能障碍和排除（tumor immune dysfunction and exclusion, TIDE）算法^[17]^预测高/低SULF1表达的LUAD患者的潜在免疫治疗反应。

### 1.14 候选靶向化合物的筛选

基于单细胞RNA测序分析中SULF1高表达成纤维细胞的差异表达基因，通过Enrichr平台（https://amp.pharm.mssm.edu/Enrichr/）Disease/Drug功能模块中的DSigDB数据库筛选药物分子^[18]^。化合物筛选阈值为P<0.05，并使用Appyters功能模块绘制火山图。

### 1.15 统计学处理

使用R软件进行统计数据分析，使用Wilcoxon检验分析方法，P<0.05为差异有统计学意义。

## 2 结果

### 2.1 IPF和LUAD中加权基因共表达模组的鉴定

本研究鉴定出25个与IPF发生密切相关的基因模组，其中9个模组与IPF发生显著相关[black (cor=0.41, P=1e-09), light green (cor=0.41, P=2e-09), saddle brown (cor=0.28, P=5e-05), dark gray (cor=0.63, P=8e-24), dark turquoise (cor=0.58, P=1e-19), dark orange (cor=0.4, P=3e-09), salmon (cor=0.28, P=5e-05), blue (cor=0.24, P=6e-04), and turquoise (cor=0.47, P=2e-12)]（图1A，图1B）。同时，鉴定出20个与LUAD密切相关的基因模组，其中8个模组与LUAD发生显著相关[dark green (cor=0.24, P=0.009), tan (cor=0.33, P=3e-04), magenta (cor=0.56, P=2e-10), midnight blue (cor=0.6, P=3e-12), blue (cor=0.68, P=1e-16), green (cor=0.95, P=7e-60), royal blue (cor=0.26, P=0.005), and green yellow (cor=0.41, P=6e-06)]（图1C，图1D）。在考虑基因数量和相关程度后，我们最终选择了IPF相关的dark gray模组（图1E）和dark turquoise模组（图1F），以及LUAD相关的green模组（图1G）和blue模组（图1H）进一步研究。

**图1:**
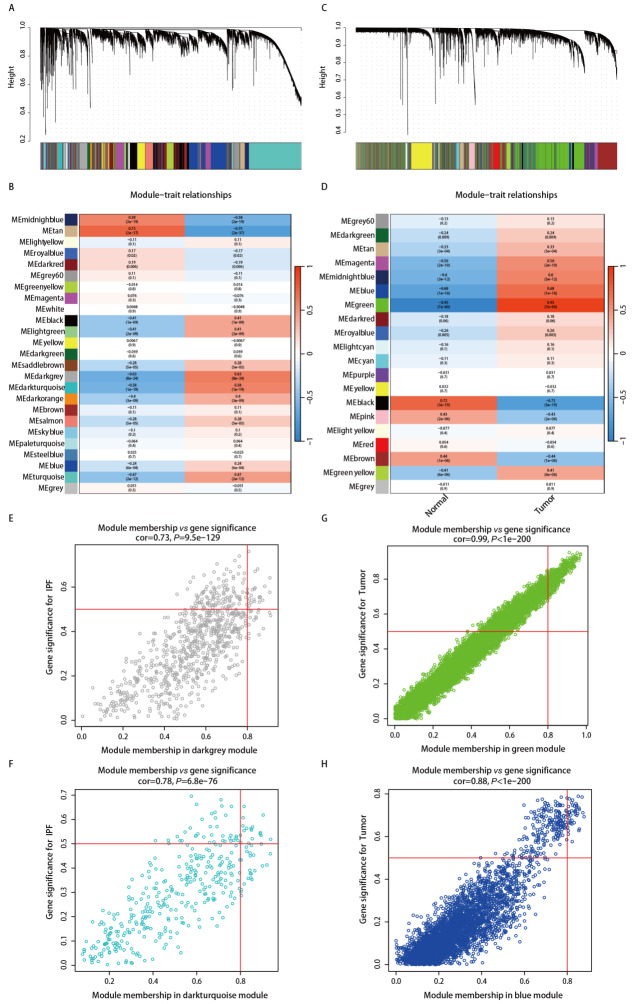
IPF和LUAD中加权基因共表达模组的鉴定。A：IPF中共表达基因的聚类树状图；B：IPF中模组-特征关系的热图；C：LUAD中共表达基因的聚类树状图；D：LUAD中模组-特征关系的热图；E-H：选定模组中基因的显著性和模组成员的散点图。

### 2.2 IPF和LUAD中共同致病基因的鉴定和功能分析

通过对4个目标模组基因取交集，获得529个共同基因（图2A）。随后，为了探索这些共同基因参与的调控通路，我们构建PPI网络（图2B），并进行功能富集分析。GO分析显示出肺部重塑相关进程的显著富集，包括胶原纤维组织过程、ECM组织、上皮间充质转化（epithelial-mesenchymal transition, EMT）调控、血管运输和肌肉收缩等（图2C）。同时还观察到了与炎细胞相关的调节通路，如白细胞滚动、细胞间黏附和磷酸肌醇-3激酶（phosphoinositide 3-kinases, PI3K）信号传导。此外，京都基因与基因组百科全书（Kyoto Encyclopedia of Genes and Genomes, KEGG）分析（图2D）显示出与ECM组织、伤口愈合、肌肉收缩、Wnt信号以及血小板激活/信号传导/聚集有关的通路富集。

**图2:**
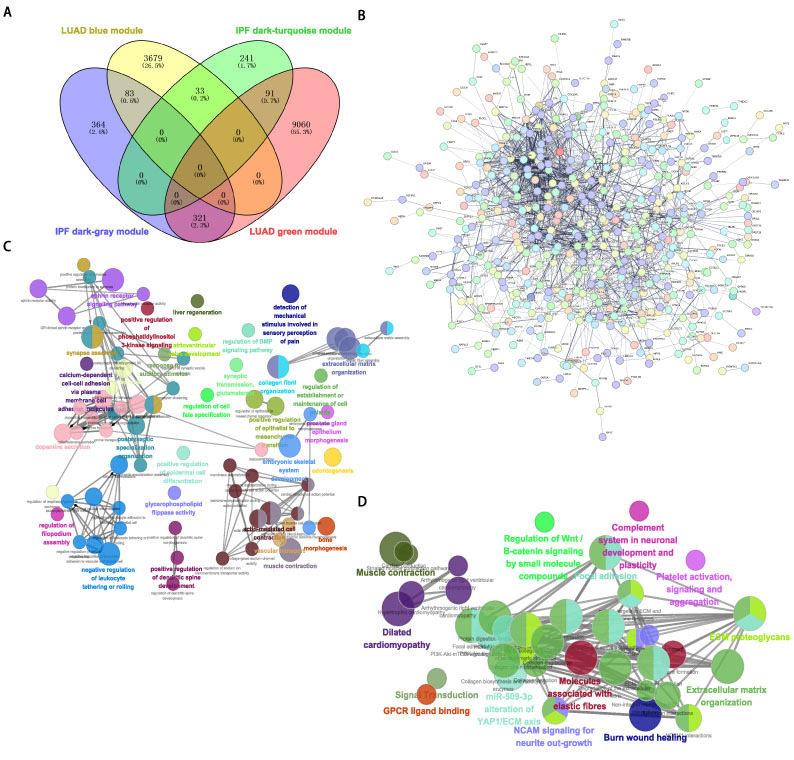
IPF和LUAD中共同基因的鉴定和功能分析。A：四个选定模组之间交集基因的Venn图；B：共同基因的蛋白相互作用网络；C：GO富集途径的网络图（Cytoscape/ClueGO）；D：KEGG富集途径的网络图（Cytoscape/ClueGO）。

### 2.3 核心共同致病基因SULF1

通过对额外的IPF和LUAD数据集进行差异表达基因（differential expressed genes, DEGs）分析（图3A，图3B），我们将DEGs与上一步得到的共同基因取交集获得的基因定义为核心共同致病基因，共14个（CDH2、CFH、COL10A1、COL1A1、COL3A1、COMP、CPA3、CXCL14、IGF1、PDLIM3、POSTN、SFRP4、SRPX、SULF1）（图3C）。我们对上述14个基因进行了生存分析，发现高表达COL1A1（图3D，图3E）、COL3A1（图3F，图3G）或SULF1（图3H，图3I）的LAUD患者总生存期（overall survival, OS）和无进展生存期（progression-free survival, PFS）均显著缩短。考虑到SULF1在LAUD中的作用尚不十分清晰，我们将SULF1作为进一步研究目标。使用GEPIA和UALCAN数据库分析发现LUAD中SULF1的mRNA表达水平显著高于正常肺组织（图3J，图3K）。进一步分析显示，与对照组相比，无论是男性还是女性，肿瘤样本中SULF1的表达均显著上调（图3L）。SULF1的表达在40岁以上LUAD患者（41-60岁组、61-80岁组和81-100岁组）均显著上调，而在21-40岁组中变化没有统计学意义（图3M）。我们还观察到在LUAD的所有分期（I、II、III和IV期）和淋巴结转移程度（N0、N1、N2或N3）中，SULF1表达均显著增加（图3N，图3O）。此外，TP53突变型和野生型LUAD患者的SULF1表达均高于对照组，并且TP53突变型患者SULF1水平显著高于野生型患者（图3P）。而SULF1的启动子甲基化水平在LUAD患者中出现显著下降（图3Q）。为了验证SULF1的蛋白表达，我们进一步对CPTAC数据库中的蛋白组学数据进行了分析。同样地，与对照组相比，LUAD组织中SULF1蛋白表达显著升高（图3R）。有趣的是，男性患者的SULF1蛋白表达明显高于女性（图3S）。对于IPF，通过另一个数据集（GSE32537）进行验证，同样发现SULF1的表达在IPF患者中相比对照组显著上调（图3T）。为了评估SULF1对IPF的诊断效能，我们构建了ROC曲线并计算了曲线下面积（area under the curve, AUC）。结果显示SULF1具有较好的（AUC值=0.948）诊断潜力（图3U）。

**图3:**
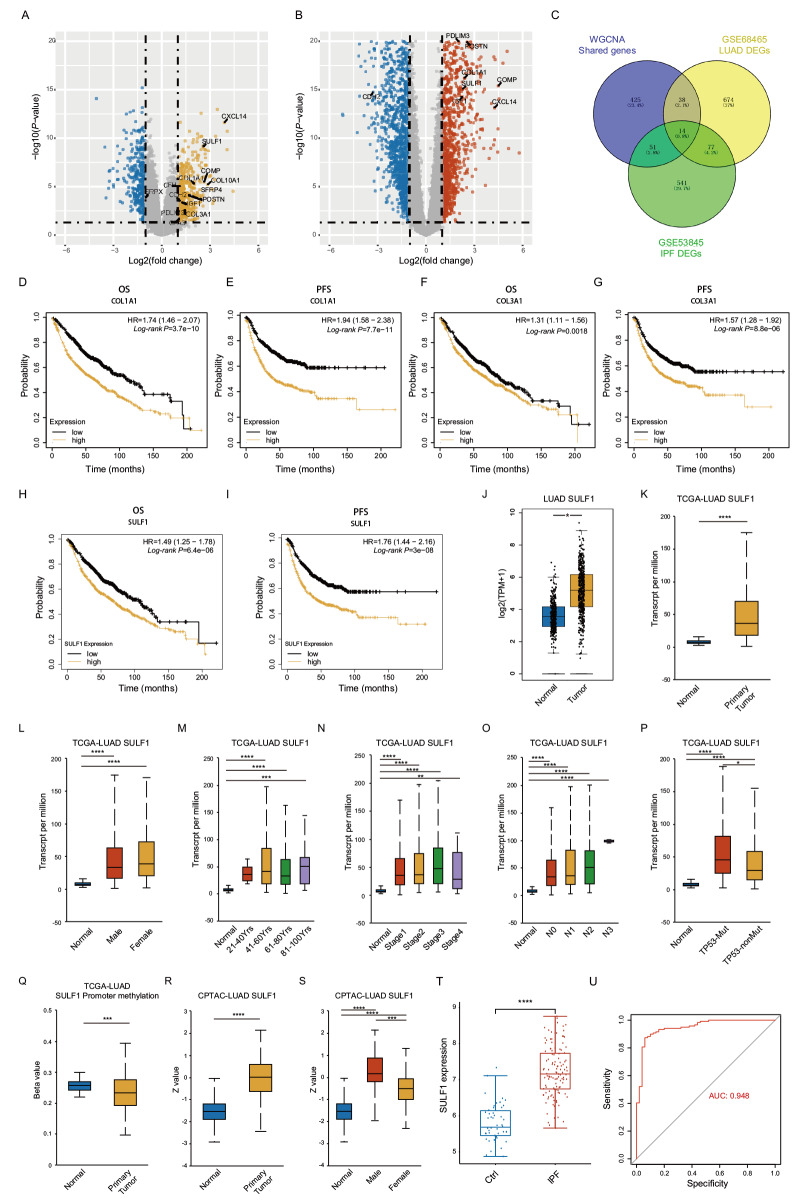
SULF1作为核心共同基因的鉴定。A：IPF数据集中差异表达基因的火山图；B：LUAD数据集中差异表达基因的火山图；C：在WGCNA共同基因和额外数据集DEGs中鉴别核心共同基因的Venn图；D-I：利用Kaplan-Meier绘图描绘OS和PFS的生存曲线；J：在GEPIA数据库中比较LUAD与正常组织中的SULF1表达；K-P：利用UALCAN数据库根据临床参数将不同组别的患者的SULF1表达进行比较；Q：基于UALCAN数据库的SULF1启动子甲基化情况；R，S：利用UALCAN数据库根据临床参数将不同组别的患者的SULF1蛋白表达进行比较；T：在GSE32537数据集中比较IPF与对照组织中SULF1表达；U：IPF中SULF1表达的ROC曲线分析。*P<0.05；**P<0.01；***P<0.001；****P<0.0001。

### 2.4 SULF1表达与LUAD遗传特征分析

为了探究SULF1表达对LUAD遗传特征的影响，我们比较了高和低SULF1表达LUAD患者之间的基因突变谱（图4A）。高SULF1表达组在TP53、TTN、CSMD3、LRP1B、COL11A1、PCLO、ADAMTS12和KEAP1基因中的突变频率显著升高。此外，高SULF1表达组还显示出更高的非整倍体水平（图4B）和肿瘤突变负荷（tumor mutational burden, TMB）评分（图4C）。DNA损伤修复分析显示高SULF1表达的样本具有更高的同源重组修复缺陷（homologous recombination deficiency, HRD）（图4D）和肿瘤内异质性（图4E）评分，而在肿瘤微卫星不稳定性评分（图4F）方面，高和低SULF1表达组之间没有发现显著差异。

**图4:**
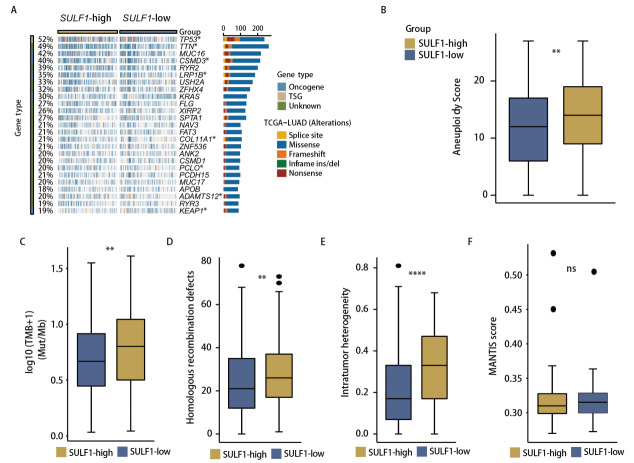
基于SULF1表达水平分层的LUAD的遗传特征。A：展示LUAD高表达和低表达SULF1组之间基因突变的瀑布图；B-F：比较LUAD高表达和低表达SULF1组之间的评分。*P<0.05；**P<0.01；****P<0.0001。

### 2.5 SULF1与LUAD肿瘤微环境分析

MCPcounter算法显示高SULF1表达患者具有多种免疫细胞的浸润，包括总T细胞、CD8^+^ T细胞、细胞毒性淋巴细胞、B细胞系、自然杀伤（natural killer, NK）细胞、单核细胞系，尤其是成纤维细胞显著增加，然而其中性粒细胞浸润减少（图5A）。抑制性免疫检查点表达分析中（图5B），高SULF1表达的LUAD患者多种抑制性免疫检查点显著上调，包括HAVCR2、TIGIT、LAG3、IDO、ADORA2A、CD276、CD274、PDCD1、PDCD1LG2、CD28、CTLA4和BTLA。此外，通过GSEA分析，我们发现高SULF1表达组在多个与免疫相关的通路中富集（图5C），如趋化因子信号传导、白细胞迁移以及B细胞和NK细胞等免疫细胞介导的免疫，这与我们从免疫浸润分析中观察到的结果一致。有趣的是，高SULF1表达组还富集于T细胞免疫负调节、耐受诱导和抑制性细胞因子信号（白介素-10）以及M2细胞因子（白介素-4和白介素-13）信号，这与我们关于抑制性免疫检查点的分析结果一致。此外，高SULF1表达组在与ECM组织和血管生成相关的通路中也显著富集，表明其在肿瘤生长和转移中的潜在作用。

**图5:**
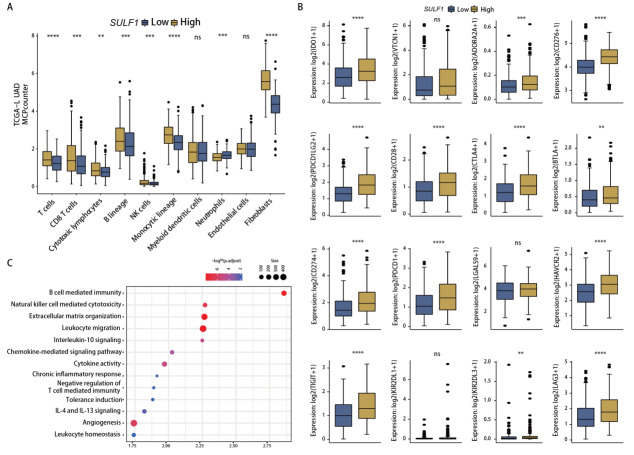
SULF1与LUAD中的肿瘤微环境。A：高表达组与低表达组之间免疫细胞浸润模式的箱线图；B：高SULF1和低SULF1组LUAD中免疫检查点抑制剂的表达；C：高表达和低表达SULF1组LUAD中差异通路的气泡图。**P<0.01；***P<0.001；****P<0.0001。

### 2.6 在单细胞水平上分析LUAD中的SULF1表达

质控后共65,722个细胞（36,597个细胞来自LUAD样本和29,125个细胞来自对照组）纳入分析，聚类为24个细胞亚群（图6A）。基于经典标记物（图6B），我们鉴定出15种主要细胞类型，包括T细胞（CD3E、IL7R）、巨噬细胞（MARCO、APOC1）、肺泡上皮细胞（EPCAM、SFTPB）、B细胞（MS4A1、CD19）、NK细胞（NKG7、KLRD1）、髓样树突状细胞（CD1C、FCER1A）、中性粒细胞（S100A12、S100A9）、纤毛细胞（FOXJ1、CAPS）、内皮细胞（VWF、PECAM1）、细支气管外分泌细胞（SCGB1A1、MUC5B）、肥大细胞（GATA2、CPA3）、成纤维细胞（PDGFRA、LUM）、浆细胞（IGKC、IGHG1）、单核细胞（FCN1、CSF1R）和浆细胞样树突状细胞（LILRA4、IL3RA）（图6C）。LUAD样本中SULF1表达显著上调（图6D），验证了前述的研究结果。随后的分析发现SULF1主要由成纤维细胞表达，且来自LUAD的成纤维细胞的表达水平高于对照组（图6E、图6F）。我们进一步提取了成纤维细胞进行亚群分析，并以1为表达量阈值将其分类为SULF1高表达成纤维细胞或SULF1低表达成纤维细胞。在LUAD中，SULF1高表达成纤维细胞数量显著多于对照组（图6G）。此外，在SULF1高和低表达的成纤维细胞之间进行的差异基因分析鉴定出118个DEGs。这些DEGs的富集分析显示它们参与了与B细胞分化调节、白细胞迁移和黏附、趋化因子信号传导、T细胞受体信号传导调控、ECM组织、伤口愈合和血管生成有关的通路（图6H）。这些发现与我们从TCGA数据集中高SULF1表达样本获得的富集结果相符。

**图6:**
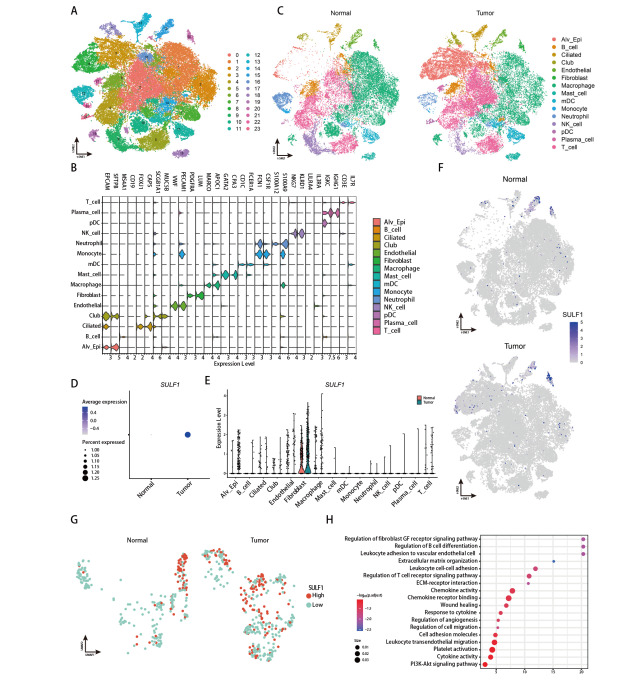
LUAD单细胞分析。A：24个细胞群聚类结果的t-SNE可视化；B：各主要细胞类型的细胞标志物的小提琴图；C：肿瘤组和对照组中15个主要细胞类型的t-SNE可视化；D：在GEPIA数据库中显示LUAD相对于正常组织的SULF1表达；E：SULF1在各种细胞类型中的表达的小提琴图；F：SULF1表达模式的t-SNE可视化；G：提取的肿瘤和对照组中的SULF1高和SULF1低细胞的UMAP可视化；H：高SULF1和低SULF1成纤维细胞之间差异通路的气泡图。

### 2.7 SULF1表达的药物敏感性分析

通过CellMiner数据库分析，我们观察到SULF1表达与LY-2606368、MPC-3100、Pelitrexol isomer A、B-7100、Pelitrexol isomer B、Malacid、Karenitecin、Methotrexate、Amonafide和Luminespib等药物的敏感性呈负相关（图7A）。与之相反地，SULF1表达与Caffeic acid、Zoledronate、HPI-1、Motesanib、IDH-C227、BMS-911543、PRN-1371、ZSTK-474、P-529和SAR-245409等药物的敏感性呈正相关（图7A）。此外， LUAD患者中高SULF1表达与更高的TIDE得分有关（图7B），表明其可能对免疫治疗的敏感性降低。最后，为了进一步筛查可能靶向SULF1高表达成纤维细胞的潜在化合物，我们使用在单细胞分析中鉴定出的118个DEGs在DSigDB数据库进行了富集分析（图7C），结果显示出Laminin、Clonidine、Loxapine、Vanadium pentoxide、Lomustine、Deptropine、N-Acetyl-L-cysteine、Acetaldehyde、Arsenenous acid和ACMC-20mvek等的化合物有潜力成为靶向SULF1高表达成纤维细胞的小分子药物。

**图7:**
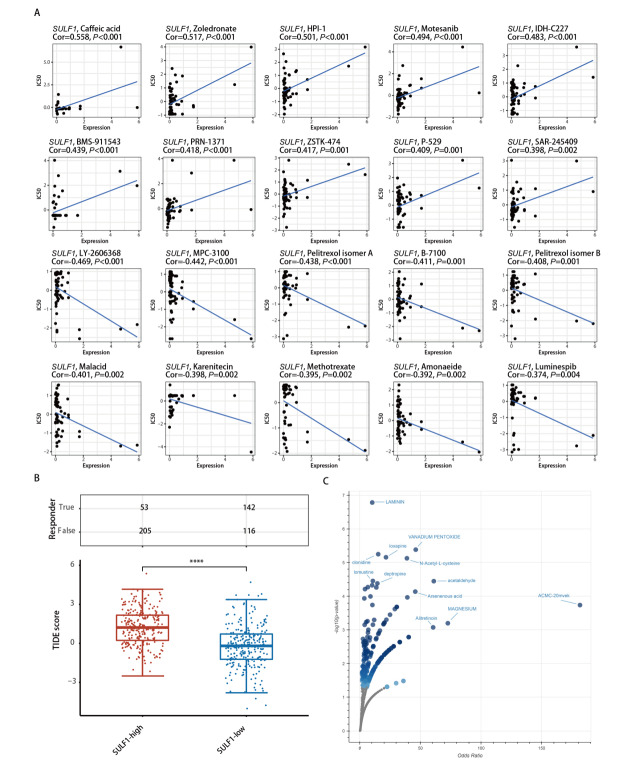
与SULF1相关的药物敏感性分析。A：利用CellMiner数据库对SULF1的药物敏感性分析；B：LUAD中高SULF1组和低SULF1组之间的TIDE评分的箱线图；C：基于Enrichr中DSigDB库的潜在小分子候选药物的火山图。****P<0.0001。

## 3 讨论

揭示IPF和LUAD之间的共同致病机制，找出肺纤维化增加肺癌风险的分子基础线索，并在此基础上探索潜在干预靶点，对于IPF合并肺癌的新药开发和个体化治疗具有重要意义。现有的研究^[4]^发现IPF和肺癌都表现出一些主要信号转导途径的异常激活。Wnt通路在IPF患者肺组织中过度激活，下游转化生长因子-β（transforming growth factor-β, TGF-β）信号失调^[1]^。Wnt途径的失调促进肿瘤进程中上皮化生、ECM沉积和EMT过程^[19]^。TGF-β1可激活细胞外信号调节蛋白激酶1和2，进而激活PI3K通路^[20]^。研究^[21]^显示PI3K信号通路的失调与纤维增生性疾病和肿瘤侵袭均密切相关。此外，酪氨酸激酶信号传导参与的细胞生长、分化、黏附和血管生成等关键调节途径，已被证实与纤维化和肿瘤的发生发展有关^[22]^。本研究通过对公共转录组学数据集进行WGCNA分析，鉴定出529个基因可能共同参与IPF和LUAD的发病。进一步的PPI和功能富集分析揭示了这些共同致病基因参与的生物学进程和信号通路，包括ECM组织、EMT、血管运输、肌肉收缩、伤口愈合、Wnt信号传导和PI3K信号转导，与之前的研究相符。同时，我们的分析还显示白细胞滚动和细胞黏附通路的富集，均是免疫应答和肿瘤进展的关键病理过程。这一发现与之前的研究^[23,24]^一致，反映这些通路在促进白细胞迁移到肿瘤微环境中，进而影响肿瘤生长、侵袭和转移方面的重要性。有趣的是，我们还发现共同致病基因在血小板激活、信号传导和聚集相关通路富集。IPF中血小板源性因子失调可引起纤维化进展^[25]^。同时这条通路在肿瘤发生发展的各个方面也发挥重要作用，如肿瘤细胞增殖、血管生成、免疫抑制，以及形成围绕肿瘤细胞的血小板“保护伞”，从而保护肿瘤躲避免疫识别，促进其转移^[26]^。此外，我们将529个共同基因与GSE53845和GSE68465中的差异表达基因进行了交集，筛选出IPF和LUAD之间的核心共同致病基因，包括CDH2、CFH、COL10A1、COL1A1、COL3A1、COMP、CPA3、CXCL14、IGF1、PDLIM3、POSTN、SFRP4、SRPX。与我们的结果部分类似的是，近期一项研究^[27]^也发现出7个IPF和LUAD共同基因，包括COL1A2、COL5A1、POSTN、CXCL13、CXCL14、CYP24A1和BMP2，并且具有良好的诊断价值。14个核心共同致病基因进一步生存分析^[28]^显示，高表达COL1A1、COL3A1或SULF1的LAUD患者OS和PFS均显著缩短。与之相符的是，Geng等^[19]^的分析发现COL1A1与肺癌免疫细胞浸润和更短的OS和PFS高度相关。另一项研究^[29]^证实上调的COL3A1与更差的肺癌预后和顺铂抵抗相关。考虑到SULF1在LAUD中的作用尚不十分清楚，本研究将SULF1作为进一步研究目标。

SULF1在ECM与细胞表面的相互作用中扮演重要角色^[30]^。早期研究^[31]^发现，TGF-β1可以上调SULF1的表达，并成为TGF-β1诱导纤维化的负调节因子。然而，新近的研究^[32]^发现，虽然SULF1起初可以保护细胞免受二氧化硅的损伤，但二氧化硅的长期暴露会通过SULF1上调增殖基因表达、增加胶原蛋白的产生和加剧纤维化。另一项近期研究^[33]^也支持SULF1促进纤维化，该研究发现敲减SULF1抑制了肌成纤维细胞激活并减少了胶原沉积，而过表达SULF1显著促进了TGF-β诱导的肌成纤维细胞激活，并在一定程度上抵消了常山酮的抗纤维化作用。本研究也发现SULF1在IPF患者肺组织上调，并对IPF具有较好的诊断价值。在肿瘤方面，早期的研究^[34]^观察到各种癌细胞系中SULF1转录本普遍低表达，因此认为其在如卵巢癌、乳腺癌和肝癌等的肿瘤中具有抑癌功能。然后，后来的研究^[35]^在多种肿瘤组织中均证实了SULF1表达的增加。在膀胱上皮癌中，高SULF1表达与更高的原发肿瘤状态与组织学分级以及更差的生存结果相关^[36]^。同样地，在胃癌中高SULF1表达与更差的OS和免疫抑制性的M2巨噬细胞浸润相关^[37]^。此外，近期的转基因小鼠和体外实验^[38]^进一步证实了SULF1促进肝癌进展和浸润的能力。本研究中，我们发现LUAD患者中SULF1的转录水平和蛋白水平均显著高于正常肺组织。进一步的分析揭示了SULF1表达与包括肿瘤分期、淋巴结转移、TP53突变状态和启动子甲基化水平等临床参数有关联。此外，我们的研究还显示了SULF1表达水平增加与较高的突变频率以及TMB和HRD等基因组异质性评分之间的正相关关系，这些异质性被认为是影响肿瘤发生和进展的重要因素^[39]^。一项研究^[40]^发现敲减SULF1不仅抑制了非小细胞肺癌细胞的增殖、迁移和侵袭，还促进了细胞凋亡，这和我们的结果都支持SULF1在促进肺癌进展方面的作用。尤为重要的是，我们还发现高SULF1表达的LUAD患者的OS和PFS更短，显示出SULF1在该疾病中的预后价值。

LAUD复杂的肿瘤微环境极大地影响着其预后。我们的分析发现SULF1高表达样本中各种免疫细胞的富集明显增加，包括B细胞、NK细胞、单核细胞和T细胞，尤其是CD8^+^细胞毒性T细胞。尽管存在显著的免疫细胞浸润，但更差的预后暗示了免疫功能抑制或受损的出现，推测这可能是由于抑制性分子的表达及免疫耐受机制的激活。符合这一推断的是，我们发现在高SULF1表达样本中，抑制性免疫检查点的表达显著增加，例如HAVCR2、TIGHIT、LAG3、IDO、ADORA2A、CD276、CD274、PDCD1、PDCD1LG2、CD28、CTLA4和BTLA。并且，通过GSEA分析我们观察到高SULF1肿瘤富集于T细胞免疫的负调节、耐受诱导、抑制性细胞因子信号传导以及免疫抑制性M2细胞因子信号传导等通路。这些结果进一步支持高表达SULF1的LUAD与抑制性微环境相关，这可能阻碍了LUAD的抗肿瘤免疫反应。此外，肿瘤相关成纤维细胞为肿瘤细胞的恶性扩张创造了有利的微环境^[41]^。与之相符的是，我们观察到在高SULF1表达水平的样本中，成纤维细胞浸润评分显著升高，同时与ECM组织和血管生成相关的通路显著富集。这表明成纤维细胞可能通过这些通路促进肿瘤的生长和转移。这些发现与一项头颈鳞癌研究^[42]^一致，该研究显示了SULF1在肿瘤组织的表达升高，并且主要来源于肿瘤相关成纤维细胞。我们的单细胞测序分析检测到LUAD样本中SULF1的表达较正常组织高，成纤维细胞为其主要表达来源，并且肿瘤相关的成纤维细胞中的SULF1水平显著高于对照组织，进一步支持了以上结果。进一步对SULF1高表达和低表达成纤维细胞的分析显示了与免疫调节、白细胞迁移、趋化因子信号传导、ECM组织和血管生成途径相关的通路富集，更加支持SULF1高表达成纤维细胞在塑造肿瘤微环境、促进免疫失调以及推动LUAD肿瘤进展方面的潜在作用。

尼达尼布已经获批非小细胞肺癌的二线治疗，而吡非尼酮在非小细胞肺癌的临床前研究中也表现出抗肿瘤活性^[43,44]^。因此，肺癌和IPF之间的共存性和部分机制的相似性为潜在药物的进一步发现提供了线索。研究^[45]^已经揭示了SULF1与卵巢癌细胞铂类耐药之间的相关性。下调SULF1降低顺铂诱导的细胞毒性，而CRISPR/Cas9筛选显示ZNF587B和SULF1的缺乏促进了卵巢癌细胞系对顺铂耐药^[46]^。本研究中，通过对CellMiner数据库的分析表明，SULF1可能与LY-2606368、MPC-3100、Pelitrexol isomer A、B-7100、Pelitrexol isomer B、Malacid、Karenitecin、Methotrexate、Amonafide和Luminespib的药物耐药性相关，同时可能增强Caffeic acid、Zoledronate、HPI-1、Motesanib、IDH-C227、BMS-911543、PRN-1371、ZSTK-474、P-529和SAR-245409在癌症治疗中的疗效，这为IPF合并LUAD患者提供了新的治疗可能性。此外，我们的研究还发现，高SULF1表达的LUAD患者可能与对免疫治疗失败相关，显示出SULF1作为LUAD中预测免疫治疗反应的生物标志物的潜力。最后，利用DSigDB库，我们对具有靶向SULF1高表达成纤维细胞能力的多种化合物进行了筛选，有助于LUAD和IPF新精准治疗策略的发现。

本研究基于生物信息学分析，其结果有待进一步利用基础和临床实验进行验证。未来SULF1的机制研究有助于阐明其如何影响LUAD和IPF疾病进展，为SULF1作为潜在的生物标志物及治疗靶点提供理论基础。

Competing interests

The authors declare that they have no competing interests.

Author contributions

Yu HJ, Li GP and Chen T conceived and designed the study. Wang JY, Lu L and He X analyzed the data. Ma LJ provided critical inputs on interpretation of the study. All authors had access to the data. All authors read and approved the final manuscript as submitted.
